# The Ki-67 proliferation index predicts recurrence-free survival in patients with dermatofibrosarcoma protuberans

**DOI:** 10.17305/bjbms.2020.5088

**Published:** 2021-04

**Authors:** Adem Deligönül, Serkan Yazici, Mine Ozsen, Sibel Kahraman Cetintas, Ulviye Yalcinkaya, Ahmet Bilgehan Sahin, Özgür Tanrıverdi, Sibel Oyucu Orhan, Birol Ocak, Erdem Cubukcu, Ramazan Kahveci, Türkkan Evrensel

**Affiliations:** 1 Department of Medical Oncology, Faculty of Medicine, Uludag University, Bursa, Turkey; 2 Department of Dermatology and Venereology, Faculty of Medicine, Uludag University, Bursa, Turkey; 3 Department of Surgical Pathology, Faculty of Medicine, Uludag University, Bursa, Turkey; 4 Department of Radiation Oncology, Faculty of Medicine, Uludag University, Bursa, Turkey; 5 Department of Medical Oncology, Faculty of Medicine, Mugla Sitki Kocman University, Mugla, Turkey; 6 Department of Plastic, Reconstructive and Aesthetic Surgery, Faculty of Medicine, Uludag University, Bursa, Turkey

**Keywords:** Dermatofibrosarcoma protuberans, Ki-67 proliferation index, disease recurrence, prognosis

## Abstract

Dermatofibrosarcoma protuberans (DFSP) is an uncommon soft tissue sarcoma that originates from the dermis or subcutaneous tissue in the skin. While its prognosis is generally favorable, disease recurrence is relatively frequent. Since morbidity after repeated surgery may be significant, an optimized prediction of recurrence-free survival (RFS) has the potential to improve current management strategies. The purpose of this study was to investigate the prognostic value of the Ki-67 proliferation index with respect to RFS in patients with DFSP. We retrospectively analyzed data from 45 patients with DFSP. We calculated the Ki-67 proliferation index as the percentage of immunostained nuclei among the total number of tumor cell nuclei regardless of the intensity of immunostaining. We constructed univariate and multivariate Cox proportional hazards regression models to identify predictors of RFS. Among the 45 patients included in the study, 8 developed local recurrences and 2 had lung metastases (median follow-up: 95.0 months; range: 5.2-412.4 months). The RFS rates at 60, 120, and 240 months of follow-up were 83.8%, 76.2%, and 65.3%, respectively. The median Ki-67 proliferation index was 14%. Notably, we identified the Ki-67 proliferation index as the only independent predictor for RFS in multivariate Cox proportional hazards regression analysis (hazard ratio = 1.106, 95% confidence interval = 1.019-1.200, *p* = 0.016). In summary, our results highlight the potential usefulness of the Ki-67 proliferation index for facilitating the identification of patients with DFSP at a higher risk of developing disease recurrences.

## INTRODUCTION

Dermatofibrosarcoma protuberans (DFSP) is an ­uncommon soft tissue sarcoma that originates from the dermis or subcutaneous tissue in the skin [1,2]. DFSP is characterized by an indolent nature and may grow to be quite large before receiving clinical attention [3]. The tumor typically originates in the dermal layer of skin [2,3] and can follow a pattern of local spread to the subcutaneous adipose tissue, muscle, fascia, and bone [4,5]. The projections of DFSP may extend beyond the margins of the clinically evident neoplasm, ultimately leading to re-growth if not adequately removed [5]. Accordingly, local recurrence rates may be as high as 60%, likely reflecting a failure to remove occult extensive tendrils [5,6]. Conversely, distant metastases are generally rare (1-4%) and long-term overall survival (OS) is favorable [2].

The main issue faced by patients with DFSP is disease recurrence – which requires repeated surgery and may result in significant morbidity [7] While certain patient and tumor characteristics (e.g., age, margin width, lesion number, and histological subtype) have been associated with an increased risk of recurrent DFSP [7,8], novel biomarkers for recurrence-free survival (RFS) hold potential to improve clinical management and provide insight into the underlying pathophysiological pathways. Immunolabeling for the Ki-67 antigen – which is typically expressed in the G2 phase of the cell cycle or during mitosis [9] – is considered a valuable tool for estimating the proportion of proliferating cells in tissue sections of various human malignancies [10,11]. Whether Ki-67 immunochemical analysis – as a means to determine the growth fraction of DFSP – may predict disease recurrence in this rare malignancy has not been entirely elucidated [5,8,12]. We, therefore, designed the current retrospective study to investigate the prognostic value of Ki-67 immunostaining with respect to RFS in patients with DFSP.

## MATERIALS AND METHODS

### Patients and follow-up

We retrospectively analyzed data from 45 adult (age >18 years) Turkish patients with biopsy-proven DFSP who were referred and followed up at the Uludag University Medical Center (Bursa, Turkey) between January 1, 1999 and September 1, 2019. Variables extracted from clinical records were age at diagnosis, sex, tumor location, tumor histotype, mitotic count, presence of necrosis, presence of metastasis at diagnosis, type of surgery, repeated surgery, surgical margins, radiotherapy (RT), and time from diagnosis. A multidisciplinary sarcoma team discussed each case and treatment decisions were taken by consensus. The surgical defect underwent primary closure whenever possible. When this approach was unfeasible because of lesion size and/or anatomic location, the closure was performed with either a skin graft or a flap. All patients were informed about the risk of potential recurrences. Follow-up consisted of ultrasound examinations at the primary lesion site and chest X-rays every 6 months for the first 5 years, and every year thereafter. Disease recurrence was defined as either local or distant recurrences (i.e., metastases). RFS and OS were defined as the time elapsed from the date of DFSP diagnosis to the date of local/distant recurrence and death, respectively. The local Institutional Review Board approved the study protocol (approval number: 2019−19/24). Due to the retrospective study design, a waiver for informed consent was obtained.

### Assessment of the Ki-67 proliferation index

**FIGURE 1:**
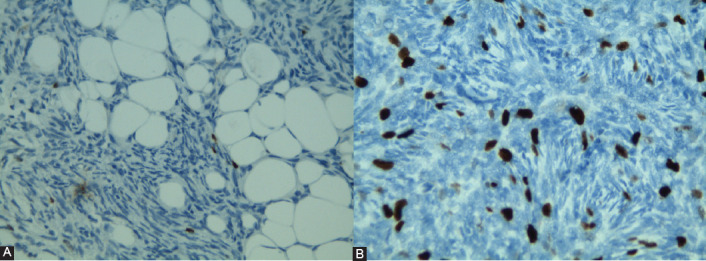
Ki-67 immunohistochemical staining of dermatofbrosarcoma protuberans has low (≤14) (A) and high (>14) Ki-67 proliferation index (B) (magnification, 10×).

Ki-67 immunostaining was carried out on paraffin-embedded archival DFSP tissue by two independent pathologists. The index was expressed as the percentage of the number of immunostained nuclei among the total number of nuclei of tumor cells regardless of the immunostaining intensity ­(possible range: 0-100%) [13]. Counts were performed in three randomly selected fields of the DFSP tissue section at 10× magnification (Figure 1). In the presence of homogeneous staining, the counting was performed in one central and two peripheral fields.

### Statistical analysis

Our study has an 87% power to detect a statistically significant difference in survival rates between patients with low versus high Ki-67 assuming an alpha error of 0.05 with a two-tailed test. We divided the patients into two groups according to the median value (14%) for the Ki-67 proliferation index (low Ki-67 ≤ 14% and high Ki-67 > 14%). The general characteristics of the study patients are presented using descriptive statistics. Kaplan–Meier plots according to the Ki-67 proliferation index were constructed and compared using the log-rank test. Univariate and multivariate Cox proportional hazards regression modeling was performed to identify predictors of RFS. A multivariate backward selection procedure was implemented. Terms in the model with a *p* < 0.20 were retained. Results are given as hazard ratios (HRs) and 95% confidence interval (CIs). Analyses were carried in SPSS, version 22.0 (IBM, Armonk, NY, USA). *p* < 0.05 (two-tailed) was used to indicate statistical significance.

## RESULTS

### Patient characteristics and clinical management

Table 1 depicts the general characteristics of the 45 study patients with DFSP. Forty-four received an extensive surgical excision, whereas one patient underwent diagnostic biopsy only. Positive and negative surgical margins were identified in 20 and 24 patients, respectively. While re-excision was performed in 14 of the 20 cases with positive margins, it was unfeasible for anatomic and/or cosmetic reasons in the remaining six. Sixteen patients received postoperative adjuvant RT. Of them, six had positive post-operative surgical margins and 10 negative but close (<2 cm) margins. The total RT dose ranged between 46.8 Gy and 62 Gy. One patient voluntarily discontinued RT. A case of radiation dermatitis was observed, and another patient developed an infection at the irradiation site. Both cases were successfully treated with local antibiotics.

**TABLE 1:**
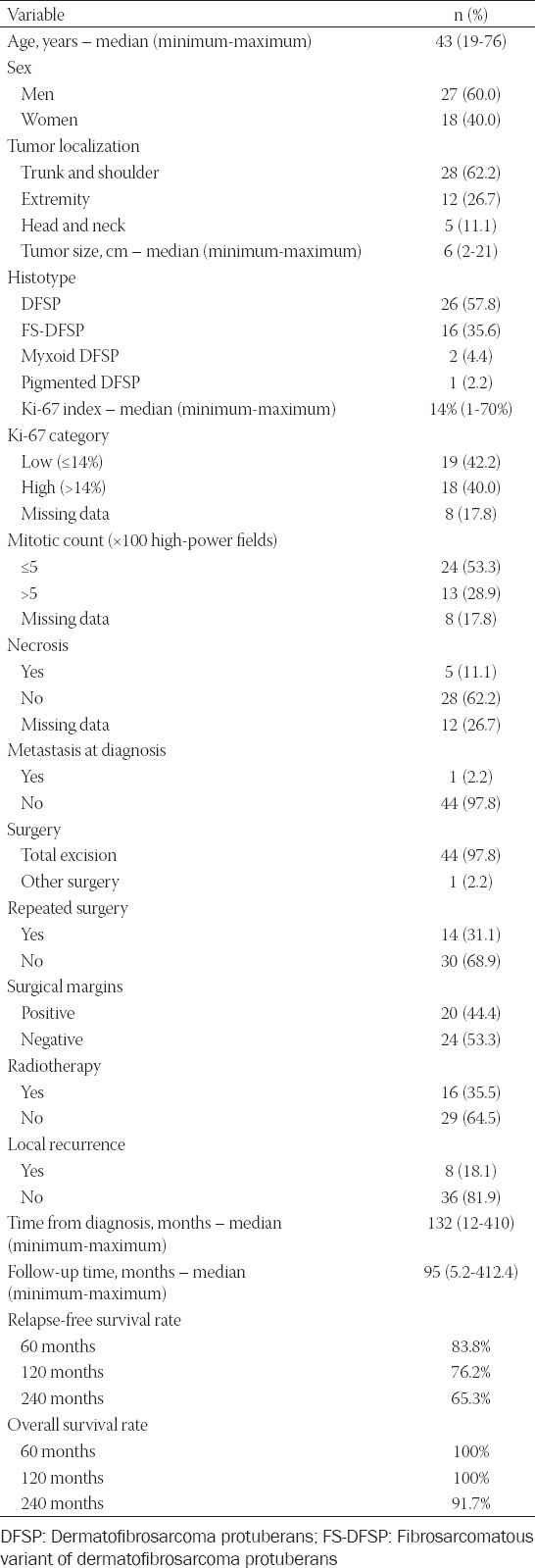
General characteristics of the 45 patients with dermatofibrosarcoma protuberans

### Disease recurrences

Patients were followed-up for a median of 95.0 months (range: 5.2-412.4 months), during which eight patients developed local recurrences and two pulmonary metastases. Patients who recurred locally underwent extensive repeated surgery. In addition, seven cases received adjuvant RT after surgery when the achievement of a sufficient margin was precluded by anatomic or cosmetic reasons. Metastasectomy was performed in one of the two cases who had distant spread to the lung; the patient survived for 5 years. Two cases developed the transformation of DFSP to fibrosarcoma. One of them had evidence of pulmonary and bone metastases on month 120 of follow-up. Transformation in the other patient occurred after two episodes of local recurrences. Systemic chemotherapy with the AIM (anthracycline, ifosfamide, and MESNA) protocol was administered as first-line treatment to all cases who experienced transformation to fibrosarcoma. A total of nine patients died (4 died of disease and 5 of other causes). The RFS rates at 60, 120, and 240 months of follow-up were 83.8%, 76.2%, and 65.3%, respectively, whereas the OS rates at the same time points were 100%, 100%, and 91.7%, respectively.

### Ki-67 proliferation index and RFS

**FIGURE 2:**
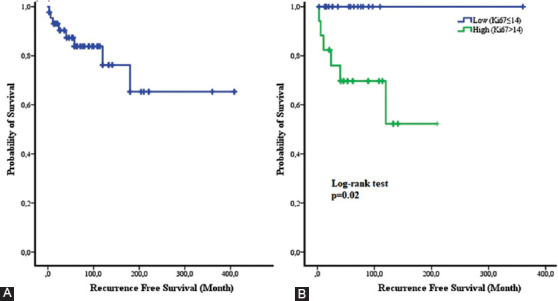
Kaplan–Meier curves depicting recurrence-free survival in the entire sample (A) and in patients stratified according to the Ki-67 proliferation index (low Ki-67 ≤ 14% and high Ki-67 >14%).

The Ki-67 proliferation index of DFSP specimens ranged from 1% to 70% (median: 14%; eight missing values). We divided patients into two groups (high vs. low) based on the optimal cutoff value for the Ki-67 score (low and high Ki-67: ≤14% and >14%, respectively). Kaplan–Meier plots revealed that RFS was significantly less favorable in patients with a high Ki-67 proliferation index (*p* = 0.002; Figure 2).

### Cox proportional hazards regression analysis

The results of univariate Cox proportional hazards regression analysis revealed that tumor size, the Ki-67 proliferation index, and the mitotic count were significantly associated with RFS (Table 2). Notably, we identified the Ki-67 proliferation index as the only independent predictor for RFS after allowance for potential confounders in multivariate analysis (HR = 1.106, 95% CI = 1.019-1.200, *p* = 0.016; Table 2).

**TABLE 2:**
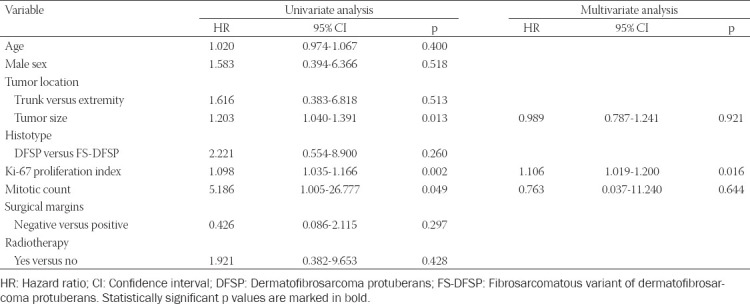
Univariate and multivariate Cox proportional hazards regression analysis for the prediction of recurrence-free survival

## DISCUSSION

This study investigated the significance of Ki-67 immunostaining in the prediction of RFS in 45 cases with DFSP enrolled at a single institution. There are two principal results from our research, which is the largest to date focusing on DFSP in Turkey. First, we confirm that patients with DFSP have a high rate of disease recurrence (26.7%) but favorable long-term OS figures. Second, we demonstrate that the Ki-67 index was the only independent predictor of RFS after allowance for potential confounders.

Of the 45 study patients, 10 patients developed disease recurrences (8 local and 2 distant). Patients with local recurrences were managed with repeated surgery with wide resection margins. Whenever anatomic or cosmetic difficulties to achieve adequate margins existed, post-operative RT [8] was given in an effort to improve local control and prevent unfavorable functional or cosmetic outcomes. Expectedly, distant recurrences were uncommon in our patients [1-3]. The rarity of DFSP and the low frequency of metastatic cases make it unlikely that prospective or randomized trials will be possible even in the future. We believe that patients with isolated distant metastasis should be individually evaluated as potential candidates for metastasectomy with curative intent. Although chemotherapy is generally considered ineffective in DFSP [14], the transformation of DFSP to fibrosarcoma should prompt systemic chemotherapy based on anthracyclines as first-line treatment.

This study provides novel information on the prognostic value of the Ki-67 index in DFSP. By multivariate analysis, this variable was identified as the only independent predictor of RFS in the study patients. This finding supports a prognostic role of cell proliferation in DFSP and has potential clinical implications not only as a risk stratification marker but also as a potential therapeutic target [15]. While there is a scarcity of published data on the prognostic value of Ki-67 immunostaining in DFSP, an association between Ki-67 and histology type has been suggested [12,16,17]. Agarwal *et al*. [12] have shown that dermatofibromas have a higher proliferation index when compared to the superficial/peripheral portion of DFSP. Using 17% as the optimal cutoff point, Du *et al*. [8] reported that patients with high Ki-67 expression had less favorable 5-year disease-free survival compared to those with low Ki-67 expression levels. Our study is the first to analyze the Ki-67 index with respect to RFS as the outcome of interest. The consistency of our findings with those of Du *et al*. [8] supports the clinical value of assessing the proliferation index Ki-67 – in addition to surgical margins – for identifying patients with DFSP who may benefit from more aggressive treatment strategies, including adjuvant RT [8].

Several caveats of our study need to be considered. First, all patients were of Turkish descent. This poses a limitation regarding the ability to generalize our conclusions, and replication in independent samples is paramount for ensuring external validity. Second, the prognostic impact of the Ki-67 with respect to OS was not specifically investigated because the number of patients who died was too limited to yield reliable statistical conclusions. Another limitation is that the Ki-67 index was the only marker investigated in the study. Consequently, we cannot rule out the possibility that other immunohistochemical markers may play a role in disease progression or RFS in patients with DFSP. Finally, retrospective studies are prone to unavoidable confounding or residual confounding on unmeasured variables.

In summary, our results highlight the prognostic ­significance of Ki-67 immunostaining for facilitating the identification of patients with DFSP at a higher risk of developing disease recurrences. Whether these cases may benefit from more aggressive treatment strategies deserves further scrutiny.
